# Effect of Weight Loss by Low-Calorie Diet on Cardiovascular Health in Type 2 Diabetes: An Interventional Cohort Study

**DOI:** 10.3390/nu13051465

**Published:** 2021-04-26

**Authors:** Shaden Melhem, Sarah Steven, Roy Taylor, Ahmad Al-Mrabeh

**Affiliations:** 1 Translational and Clinical Research Institute, Magnetic Resonance Centre, Newcastle University, Newcastle upon Tyne NE4 5PL, UK; s.s.r.melhem@sms.ed.ac.uk (S.M.); Sarah.Steven@mft.nhs.uk (S.S.); roy.taylor@ncl.ac.uk (R.T.); 2 Centre for Cardiovascular Science, University of Edinburgh, Edinburgh EH16 4TJ, UK; 3 Manchester Diabetes & Endocrinology Centre, Manchester Royal Infirmary, Manchester M13 9WL, UK

**Keywords:** Type 2 diabetes, weight loss, remission, cardiovascular disease, biomarkers

## Abstract

Cardiovascular disease (CVD) remains a major problem for people with type 2 diabetes (T2DM), and the leading cause of death worldwide. We aimed to determine cardiovascular benefits of weight loss with or without remission of diabetes, and to assess utility of plasma biomarkers. 29 people with T2DM were studied at baseline and after dietary weight loss. Change in plasma adipokines and lipid related markers was examined in relation to weight loss, diabetes remission, 10-year cardiovascular risk (QRISK), and duration of diabetes. QRISK decreased markedly after weight loss (18.9 ± 2.2 to 11.2 ± 1.6%, *p* < 0.0001) in both responders and non-responders, but non-responders remained at higher risk (15.0 ± 2.0 vs. 5.8 ± 1.6%, *p* < 0.0001). At baseline, plasma GDF-15 was higher in longer diabetes duration (1.19 ± 0.14 vs. 0.82 ± 0.09 ng/mL, *p* = 0.034), as was the QRISK (22.8 ± 2.6 vs. 15.3 ± 3.4%, *p* = 0.031). Leptin, GDF-15 and FGF-21 decreased whereases adiponectin increased after weight loss in responders and non-responders. However, the level of FGF-21 remained higher in non-responders (0.58 [0.28–0.71] vs. 0.25 [0.15–0.42] ng/mL, *p* = 0.007). QRISK change correlated with change in plasma VLDL1-TG (r = 0.489, *p* = 0.007). There was a positive correlation between rise in HDL cholesterol and the decrease in leptin (r = 0.57, *p* = 0.001), or rise in adiponectin (r = 0.58, *p* = 0.001) levels. In conclusion, weight loss markedly decreases cardiometabolic risk particularly when remission of diabetes is achieved. Leptin, adiponectin, GDF-15 and FGF-21 changes were related to weight loss not remission of diabetes. Normalization of 10-year cardiovascular risk and heart age is possible after substantial dietary weight loss and remission of T2DM.

## 1. Introduction

Cardiovascular disease (CVD) remains a major problem for people with type 2 diabetes mellitus (T2DM), and the leading cause of death worldwide [1,2,3]. Recognition that substantial dietary weight loss is feasible and effective in achieving lasting remission of T2DM [4,5,6,7] raises the possibility that clinically useful decrease in cardiovascular risk may follow. This is required to be defined, and the relationship of change in CVD risk to change in plasma biomarkers is also of importance.

Non-alcoholic fatty liver disease (NAFLD) increases hepatic triglyceride export via very low-density lipoprotein (VLDL), and this is related to the pathogenesis of T2DM [6,8,9]. These abnormalities in lipid metabolism markedly increase CVD risks [3,10,11]. Remission of T2DM by weight loss is associated with normalization of liver fat and hepatic VLDL1-TG export [4,5,6], and the changes in plasma biomarkers on CVD risk requires to be established after remission.

The associations of leptin, adiponectin, and fibroblast growth factor 21 (FGF-21) with T2DM and CVD are not fully understood [12,13,14]. Recently, the role of growth and differentiation factor 15 (GDF-15) on T2DM and CVD has been highlighted [15,16,17]. However, the effects of diabetes duration, weight loss, and separately remission of diabetes on plasma levels of these markers have not been yet established in relation to cardiovascular health. Leptin resistance in obesity and T2DM could potentially affect CVD [9,14]. However, there is conflicting information whether leptin is a marker for CVD in diabetes or not [18,19,20]. Plasma leptin-to-adiponectin ratio is considered a marker of atherogenicity in T2DM [12,20]. Obesity is an independent risk factor for T2DM and CVD even in children, and weight loss is effective in reversing diabetes and would probably decrease the CVD risks [21,22].

The Counterbalance study used a very low-calorie liquid diet to achieve around 15% weight loss in a group of overweight people with short (<4 years) or long (>8 years) duration T2DM [4]. Weight was then maintained steady over 6 months. This allows quantification of change in the 10-year cardiovascular risk score for the first time in relation to remission of type 2 diabetes. The weight loss with or without remission of diabetes provides a unique setting to study the interaction of plasma biomarkers with plasma lipids and cardiometabolic state.

## 2. Materials and Methods

### 2.1. Participants and Recruitment

Thirty individuals with T2DM of short (<4 years) or long duration (>8 years) followed a very low-calorie diet (VLCD) for 8 weeks followed by a stepwise return to isocaloric diet and weight maintenance for 6 months. The primary aim of the study was to achieve remission of diabetes, and the main outcomes were reported previously [4]. This paper reports change in CVD risk after weight loss in relation to change of major lipid-related plasma biomarkers. Participants were 25–80 years of age with BMI of 27–45 kg/m^2^. All anti-diabetic drugs were discontinued at the start of the study. Only one participant was excluded for failing to meet the 1-week weight loss target, hence 29 participants completed the study (15F/14M).

### 2.2. Weight Loss Intervention

Weight loss was induced by VLCD as liquid formula providing 624 kcal/day (43% carbohydrate, 34% protein and 19.5% fat; Nestlé, UK). In addition, up to 240 g of non-starchy vegetables were consumed per day (total energy intake was 624–700 kcal/day). Participants were encouraged to drink at least two litres of calorie-free beverages per day and to maintain their habitual level of physical activity. Participants were excluded if they were unable to achieve weight loss targets of 3.8% body weight at week one of the VLCD or 9.3% at week four as previously demonstrated [22]. During the weight maintenance phase, participants were supported by an individualized programme based on goal setting, action planning and barrier identification. Adherence to the dietary intervention was maximized by providing one-to-one support on weekly basis by telephone, e-mail, text message, or face-to-face contact [4]. The primary goal of this phase was to prevent weight regain by individualized dietary advice guided by weight trajectory [4]. Anthropometric assessments and blood samples to measure plasma metabolites were carried out at baseline, after return to normal diet (10 weeks), and after 6 months weight maintenance.

### 2.3. Abdominal, Intra-Organ, and Total Body Fat Content

Magnetic resonance data were acquired based on 3-point Dixon method as using a 3T scanner (Philips, The Netherlands) as previously described [4,22,23]. Intrapancretic fat was evaluated from three regions of interest on two image slices of pancreas using MR-opsy method [23,24]. MR data analysis were carried out blinded to the participants’ details and time point. A separate 3-point Dixon MR acquisition was obtained at L4-5 to assess abdominal fat areas. Subcutaneous (SAT) and visceral (VAT) fat areas were separated by watershed method using ImageJ (National Institutes of Health, Bethesda, Rockville, MD, USA), and total fat was measured using Bodystat1500 (Bodystat Ltd., Isle of Man, UK) as previously described [4].

### 2.4. Cardiovascular Risk

The QRISK^®^3 algorithm developed specifically for UK population was used to assess the 10-year risk of developing CVD and to evaluate the theoretical heart age [25]. QRISK score was derived from imputing the following parameters: age, sex, ethnicity, height, weight, systolic blood pressure, smoking status, diabetes status, total/HDL cholesterol ratio, and the use of anti-hypertension medication. QRISK assessment was carried out at baseline, 2 months, and 6 months after intervention. People who achieved remission of diabetes (responders: HbA1c < 48 mmol/mol [6.5%] and fasting blood glucose < 7.0 mmol/L off all anti-diabetes medication) were considered diabetes free while entering data to measure the QRISK.

### 2.5. HOMA Index

Homeostasis Model Assessment of insulin resistance (HOMA-IR) was calculated using the HOMA2 Calculator (University of Oxford, Oxford, UK).

### 2.6. Lipoprotein Analysis

VLDL1-TG production rate of the liver was measured based on a non-isotopic method of Intralipid infusion as previously described [4,6,26]. VLDL1 lipoprotein fraction was separated from plasma be ultracentrifugation [6] and TG content was determined used the standard methods (Roche Diagnostics, Burgess Hill, UK).

### 2.7. Biomarkers Analysis

Plasma levels of leptin, adiponectin, GDF-15, and FGF-21 were measured by ELISA using commercial kits (R&D Systems, Minneapolis, MN, USA) with coefficients of variation of 4.8%, 4.2%, 3·1% and 4·3%, respectively. Total plasma ApoB and ApoE were measured by commercial ELISA kits (Mabtech, Stockholm, Sweden) with coefficients of variation of 4.4%, and 4.7%, respectively.

### 2.8. Analytical Procedures

Plasma hormones and metabolites were measured at a Clinical Pathology Accredited laboratory (Newcastle upon Tyne Hospital NHS Foundation Trust, Department of Clinical Biochemistry) using standard methods [4]. Glucose was measured using a Yellow Springs glucose analyser (Yellow Springs Inc., Yellow Springs, OH, USA). HbA1c was measured by HPLC (Tosoh Bioscience, Griesheim, Germany) and insulin was measured by ELISA (Mercodia Inc., Uppsala Sweden). Total TG and cholesterol levels were measured using standard methods (Roche Diagnostics, Burgess Hill, UK).

### 2.9. Statistical Analysis

Data were obtained from clinical follow up, MRI studies, routine laboratory tests, and additional analyses of biomarkers. All data compared between responders who achieved non-diabetic glucose control and non-responders who did not. The primary data on pathophysiological observations has been published [4], and the present report concerns new analyses of cardiovascular CVD risk scores and plasma biomarkers. The study was powered based on expected change in first phase insulin response after remission of diabetes as described previously described [4]. Data are presented as mean ± SEM or median (IQR) based on distribution. Statistical analyses were carried out using Student’s paired and 2-sample t-test, Mann–Whitney U, and Wilcoxon Rank. Spearman Rank or Pearson were used as appropriate for assessing correlation. All statistical analysis were carried out using Minitab 17 (Minitab Inc., State College, PA, USA).

## 3. Results

### 3.1. Baseline Characteristics of Participants

Gender balance, body weight, and plasma total triglycerides were similar between both groups at baseline (Table 1). Responders were younger (52.0 ± 2.9 vs. 59.9 ± 2.1 years, *p* = 0.039) with lower total fat percentage than non-responders (36.2 ± 1.9 vs. 42.6 ± 2.2%, *p* = 0.033). There were no significant differences in intraorgan fat, total cholesterol, leptin, and adiponectin between both groups (Table 1). HDL cholesterol was marginally lower in the responder group (1.1 ± 0.1 vs. 1.4 ± 0.1 mmol/L, *p* = 0.050). There were no significant differences between responders and non-responders at baseline in estimated 10-year risk score of CVD (15.3 ± 3.4 vs. 21.4 ± 2.9%, *p* = 0.18) nor in estimated heart age (67.8 ± 2.9 vs. 74.6 ± 1.9 years, *p* = 0.067).

### 3.2. Effect of Weight Loss on CVD Risk

**Figure 1:**
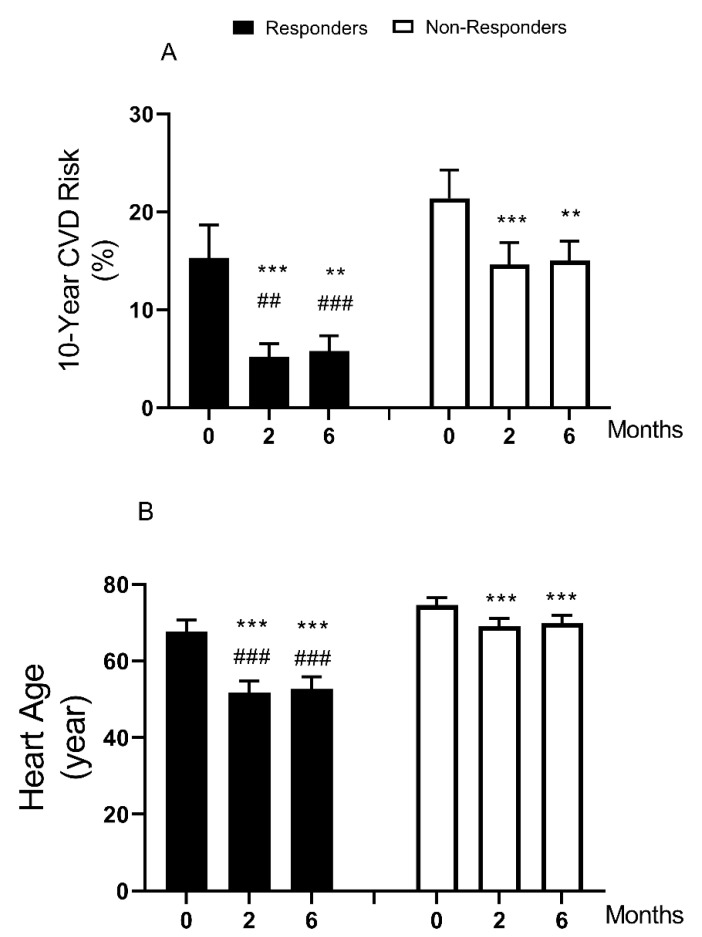
Change in cardiovascular disease (CVD) risk and heart age after weight loss and remission of type 2 diabetes. Data were presented as mean ± SEM. A: 10-year CVD Risk, B: Heart Age. There were 29 participants (12 responders/17 non-responders) and they were studied at baseline, 2, and 6 months post-weight loss. ** *p* < 0.01, *** *p* < 0.001 (vs. 0 month). ## *p* < 0.01, ### *p* < 0.001 (vs. non-responders).

The 10-year cardiovascular risk decreased markedly in the whole group during weight loss (18.9 to 11.2%, *p* < 0.0001). A decrease was seen in both responders (15.3 ± 3.4 to 5.8 ± 1.6%, *p* < 0.0001) and non-responders (21.4 ± 2.9 to 15.0 ± 2.0%, *p* < 0.0001), Figure 1/Table 1. Estimated heart age also decreased after weight loss in the whole group (71.8 ± 1.8 to 62.8 ± 2.4 years, *p* < 0.0001): responders (76.8 ± 2.9 to 52.7 ± 3.3 years, *p* < 0.0001) and non-responders (74.6 ± 1.9 to 69.9 ± 2.0 years, *p* < 0.0001), Table 1/Figure 1. There was a significantly greater improvement in responders compared with non-responders (−15.1 ± 0.9 vs. −4.6 ± 0.9 years, *p* < 0.0001). Notably, the difference between actual and estimated heart age was high at baseline for the whole group (56.7 ± 1.8 vs. 71.8 ± 1.8 years, *p* < 0.0001), and this effectively normalized after weight loss with remission of diabetes (52.0 ± 2.9 vs. 52.7 ± 3.3 years).

At baseline, there was a correlation between CVD risk and visceral fat volume (r = 0.75, *p* < 0.0001, Figure S1A), and this remained similar after weight loss (r = 0.51, *p* = 0.005). There was no such relationship between subcutaneous fat and CVD risk (r = −0.24, *p* = 0.21) or after weight loss (r = 0.03, *p* = 0.90).

### 3.3. Weight Loss Effect on GDF-15 and FGF-21

**Figure 2:**
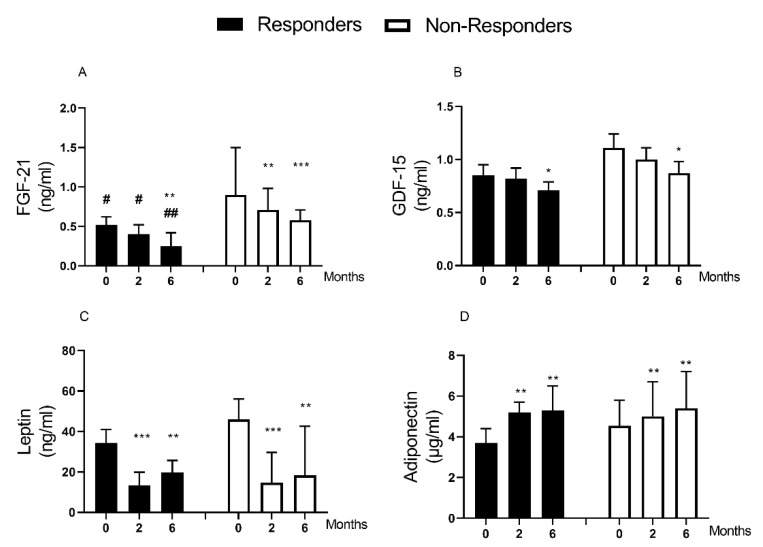
Change in plasma biomarkers after weight loss and remission of type 2 diabetes. Data are presented as median (IQR) except for GDF-15 (mean ± SEM). (**A**): FGF-21, (**B**): GDF-15, (**C**): Leptin, (**D**): Adiponectin. There were 29 participants (12 responders/17 non-responders) studied at baseline, 2, and 6 months post-weight loss. FGF-21 Data for one responder is missing (n = 11). * *p* < 0.05 vs. 0 month, ** *p* < 0.01 vs. 0 month, *** *p* < 0.001 vs. 0 month. # *p* < 0.05 vs. non-responders, ## *p* < 0.05 vs. non-responders.

Following weight loss, there were major decreases in plasma FGF-21 (0.76 [0.50–1.10] to 0.44[0.22–0.59] ng/mL, *p* < 0.0001) and GDF-15 (1.00 ± 0.09 to 0.80 ± 0.07 ng/mL, *p* = 0.004). FGF-21 decreased similarly in responders and non-responders (0.52 [0.37–0.62] to 0.25[0.15–0.42] ng/mL, *p* = 0.005, and 0.90 [0.74–1.50] to 0. 58 [0.28–0.71] ng/mL, *p* < 0.0001, respectively, Figure 2A). FGF-21 level was lower in responders compared with non-responders at baseline (0.52 [0.37–0.62] vs. (0.52 [0.37–0.62] ng/mL, *p* = 0.01), and this remained so after weight loss (0.25 [0.15–0.42] vs. 0.58 [0.28–0.71] ng/mL, *p* = 0.007).

GDF-15 decreased in responders (0.85 ± 0.10 to 0.71 ± 0.08 ng/mL, *p* = 0.017) and non-responders (1.11 ± 0.13 to 0. 87 ± 0.11 ng/mL, *p* = 0.034), Figure 2B. There was no significant difference in baseline GDF-15 levels between responders and non-responders (*p* = 0.132).

**Figure 3:**
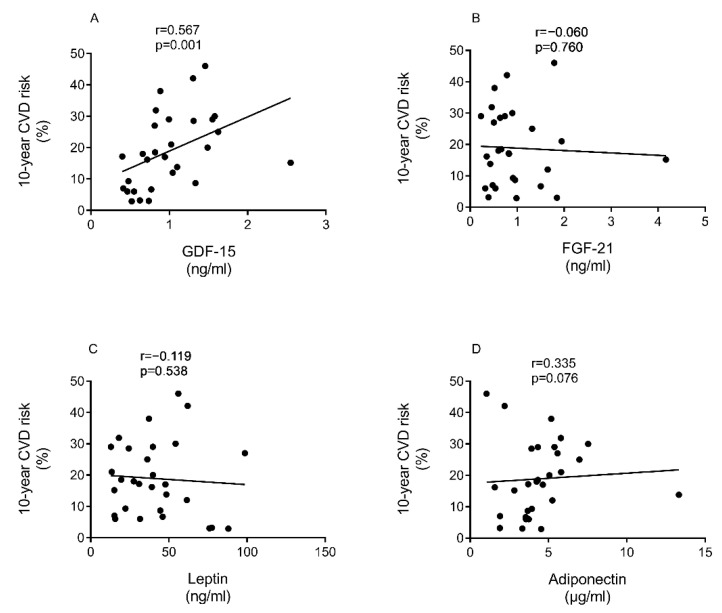
Correlation between CVD risk and plasma biomarkers at baseline in terms of 10-year CVD risk with GDF-15 (**A**), FGF-21 (**B**), Leptin (**C**), and Adiponectin (**D**). Twenty-nine participants (12 responders/17 non-responders) were included. Spearman Rho/Pearson Correlations were used as appropriate. Correlations were done at baseline, there was no change in relationship over time.

At baseline, there was a correlation between CVD risk and GDF-15 levels (r = 0.57, *p* = 0.001, Figure 3A), and this remained after weight loss (r = 0.55, *p* = 0.02). However, there was no relationship between extent of change of GDF-15 and the observed improvement in CVD risk after weight loss (Figure S2A). There was no relationship between FGF-21 and CVD risk at baseline (r = −0.06, *p* = 0.76, Figure 3B) or after weight loss (r = 0.01, *p* = 0.96).

### 3.4. Weight Loss Effect on Leptin and Adiponectin

Leptin levels decreased (39.1 [22.2–54.3] to 18.3 [8.7–36.3] ng/mL, *p* < 0.0001) whereas adiponectin increased 4.2 [3.6–5.3] to 5.4 [3.9–7.2] µg/mL, *p* < 0.0001) after weight loss. The fall in leptin was similar in responders and non-responders (34.4 [22.2–41.0] to 19.8 [8.5–25.7] ng/mL, *p* = 0.003; and 46.1 [22.2–56.1] to 18.3 [10.3–42.6] ng/mL, *p* = 0.002, respectively, Table 1/Figure 2C). Adiponectin increased similarly in responders and non-responders (3.7 [3.0–4.4] to 5.3 [3.9–6.5] µg/mL, *p* = 0.01, and 4.5 [3.7–5.8] to 5.4 [4.2–7.2] µg/mL, *p* = 0.007, respectively, Table 1/Figure 2D).

There was no relationship between baseline levels or the extent of change of leptin or adiponectin and the observed improvement in CVD risk after weight loss (Figure 3C,D; Figure S2C,D). The ratio of leptin to adiponectin (index of adipose tissue dysfunction) decreased after weight loss (7.9 [5.3–13.0] to 3.9 [1.6–8.7] ng/µg, *p* < 0.0001). This decreased similarly in responders (7.9 [6.4–14.8] to 3.9 [1.1–7.6], ng/µg *p* = 0.003), and non-responders (8.3 [5.2–13.0] to 3.9 [1.6–9.7] ng/µg, *p* < 0.0001, Table 1).

**Figure 4:**
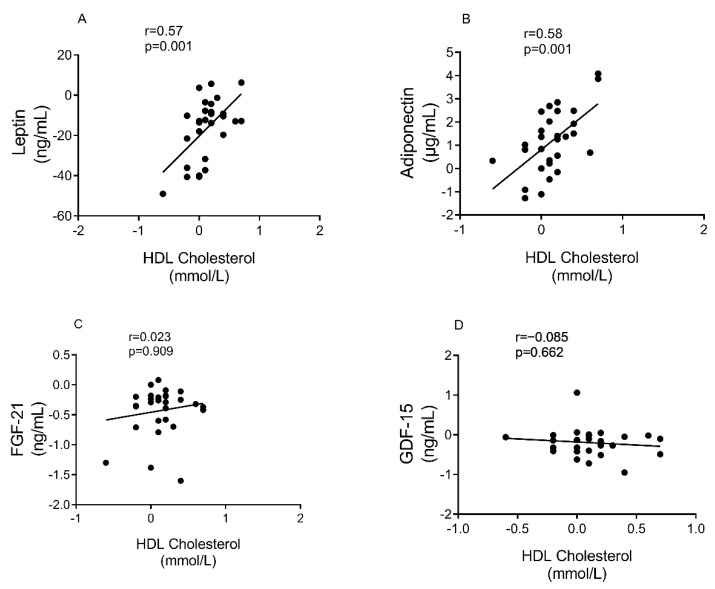
Correlation between changes in HDL cholesterol and plasma biomarkers. (**A**): Leptin, (**B**): Adiponectin, (**C**): FGF-21, and (**D**): GDF-15. 29 participants (12 responders/17 non-responders) were included. Spearman Rho /Pearson were used to assess correlation on the changes from baseline at 6 months.

There was a positive correlation between the rise in HDL cholesterol and the decrease in leptin (r = 0.57, *p* = 0.001), and rise in adiponectin (r = 0.58, *p* = 0.001) levels, Figure 4A,B). However, there was no such relationships between HDL cholesterol and FGF-21(r = 0.02, *p* = 0.91) or GDF-15 (r = −0.09, *p* = 0.66), Figure 4C,D.

### 3.5. Weight Loss Effect on Lipoprotein Profile

Hepatic VLDL1-TG production decreased equally in responders (411.0 ± 49.5 to 257.8 ± 38.9 mg/kg/day, *p* = 0.001) and non-responders (437.2 32.4 to 308.1 ± 30.4 mg/kg/day, *p* = 0.003), Table 1. This was accompanied by a concurrent decrease in liver fat content in responders (10.6 [7.0–16.0] to 1.7 [1.6–2.2]%, *p* < 0.0001) and non-responders (7.3 [5.4–9.8] to 2.0 [1.7–2.6]%, *p* < 0.0001), Table 1. Plasma concentration of VLDL1-TG and VLDL1-TG pool also decreased in both groups (VLDL1-TG: 0.60 [0.44–0.89] to 0.36 [0.25–0.45] mmol/L, *p* = 0.008, and 0.43 [0.33–0.63] to 0.28 [0.17–0.42] mmol/L, *p* = 0.008, respectively; VLDL1-TG pool: 2322.5 [1496.4–3248.0] to 1038.4 [755.8–1343.2] mg, *p* = 0.004, and 1404.3 [929.3–2509.3] to 607.8 [513.5–1493.5] mg, *p* = 0.005, respectively).

There was a positive correlation between change in CVD risk and change in plasma VLDL1-TG concentration (r = 0.489, *p* = 0.007). Fasting VLDL1-TG tended to correlate negatively with adiponectin levels at baseline (r=−0.31, *p* = 0.11), and this became significant after weight loss (r = −0.49, *p* = 0.007).

There was no significant change in ApoB level after weight loss in both responders (3.5 ± 1.1 to 3.9 ± 1.2 mg/mL, *p* = 0.59) and non-responders (1.5 ± 0.3 to 1.5 ± 4 mg/mL, *p* = 0.99), Table 1. The difference was significant between responders and non-responders at 6 months (3.9 ± 1.2 vs. 1.5 ± 4 mg/mL, *p* = 0.02). ApoE level also decreased after weight loss in both responders (14.2 [9.3–17.0] to 5.1 [4.2–12.9] µg/mL, *p* = 0.004) and non-responders (7.0 [5.6–10.4] to 5.1 [3.3–8.6] µg/mL, *p* = 0.03).

There was a correlation between the change in QRISK score and ratio of VLDL1-TG/ApoB and (r = 0.42, *p* = 0.02). The ratio of non-HDL cholesterol/ApoB as an index of the size of the LDL particle was not different between responders and non-responders at baseline (2.3 ± 0.54 vs. 3.0 ± 0.35 mmol/L/mg/mL, *p* = 0.31). However, it became largely lower in responders at 6 months (1.6 ± 0.36 vs. 2.9 ± 0.41 mmol/L/mg/mL, *p* = 0.001).

### 3.6. Effect of Diabetes Duration on Change in Plasma Biomarkers and CVD Risk after Weight Loss

Data for short and long duration groups are presented in Table S1. The gender ratio was similar in short and longer duration groups, as were body weight, total fat and total triglycerides at baseline. People in the short duration group were around 10 years younger (52.1 ± 2.6 vs. 61.6 ± 2.0 years, *p* = 0.007) with higher fasting insulin and lower fasting glucose compared to those in the longer duration group (17.4 [11.5–22.4] vs. 7.0 [5.7–11.6] mU/L, *p* = 0.006 and 9.6 ± 0.7 vs. 13.4 ± 0.8 mmol/L, *p* = 0.001, respectively). There were no significant differences in intraorgan fat, total cholesterol, leptin, adiponectin, and FGF-21 between both groups. However, GDF-15 plasma levels were lower in the short duration diabetes group (0.82 ± 0.09 vs. 1.19 ± 0.14 ng/mL, *p* = 0.034, and HDL cholesterol (1.1 ± 0.1 vs. 1.4 ± 0.1 mmol/L, *p* = 0.043). The estimated 10-year risk scores of CVD (QRISK) were lower in short than long duration group (15.3 ± 3.4 vs. 22.8 ± 2.6%, *p* = 0.031) and average heart age was lower (68.2 ± 2.6 vs. 75.6 ± 2.0 years, *p* = 0.031). In the short duration group after weight loss, QRISK score improved dramatically and heart age became close to actual age (from 68.2 ± 2.6 to 56.3 ± 3.l years at 6 months; actual age 52.6 ± 2.9 years plus 8 months study duration; Table 1). In the long duration group after weight loss, both CVD risk score and heart age improved but remained substantially raised (Table S1).

Body weight decreased similarly in short and long duration groups after intervention (−14.3 ± 1.5 vs. −12.3 ± 1.2 kg, *p* = 0.45) with similar decrease in the volumes of subcutaneous (−68.5 ± 18.3 vs. 67.0 ± 11.0 cm^2^, *p* = 0.81) and visceral fat (−100.3 ± 16.6 vs. −94.7 ± 14.6 cm^2^, *p* = 0.78). Leptin decreased and adiponectin increased, respectively, in the short (39.1 [29.258.8] to 20.5 [11.5–31.6] ng/mL, *p* < 0.0001, and 3.7 [3.5–4.4] to 5.3 [3.8–6.1] μg/mL, *p* = 0.003) and long duration groups (38.0 [15.9–47.4] to 16.0 [9.1–36.3]ng/mL, *p* = 0.006, and 4.9 [3.7–5.7] to 5.9 [4.2–7.4] μg/mL, *p* = 0.01). Similarly, GDF-15 and FGF-21 decreased in both short duration (0.82 ± 0.09 to 0.69 ± 0.06 ng/mL, *p* = 0.015, and 0.64 [0.47–0.97] to 0.41 [0.25–0.56] ng/mL, *p* = 0.001, respectively), and long duration groups (1.19 ± 0.14 to 0.92 ± 0.13 ng/mL, *p* = 0.017, and 0.79 [0.51–1.32] to 0.49 [0.22–0.71] ng/mL, *p* = 0.002, respectively).

Hepatic VLDL1-TG production was not different at baseline and decreased after weight loss in short (428.6 ± 39.2 to 295.3 ± 37.5 mg/kg/day, *p* < 0.0001) and long duration groups (425.0 ± 38.9 to 278.6 ± 30.7 mg/kg/day, *p* = 0.011. Similarly, VLD1-TG pool decreased in both groups (2370.9 [1082.5–3248.0] to 1236.5 [563.0–1432.8] mg, *p* = 0.001) and 1404.3 [1223.6–1951.5] to 696.9 [521.0–1061.8] mg, *p* = 0.025, respectively). ApoB level was similar and did not change significantly after weight loss in both groups (Table S1). Short duration group had higher baseline level of ApoE (15.6 [9.6–17.6] vs. 6.3[4.8–9.3] µg/mL, *p* = 0.007).

## 4. Discussion

Weight loss induced remission of T2DM by low-calorie diet brought about 62% decrease in 10-year CVD risk and normalization of heart age. Similar weight loss without remission decreased CVD risk by 30% (Table 1/Figure 1). To set this in context, the risk reduction achieved by statin therapy is around 22% [27]. Abnormal lipid metabolism is a hallmark of T2DM and a major determining factor for cardiovascular health [8,9]. Weight loss by calorie restriction can correct the derangement in lipid metabolism and achieve lasting remission of diabetes [4,5,6,7,22,28]. The extent of improvement of CVD risk after weight-loss induced remission of T2DM has not previously been reported. Imbalance in adipokines levels in T2DM is related to over expansion and dysfunction in adipose tissues and this is likely to be associated with CVD risks [9,14].

The observed changes in 10-year cardiovascular risk after dietary weight loss are remarkable. Given the atherogenic nature of liver derived lipoproteins (VLDL) and their remnants [11], the improvement of CVD is likely to be largely attributable to the change in lipoprotein profile whilst recognising other factors [29]. The risk of incident T2DM has been reported to be determined by the size of the lipoprotein particles rather than lipid content and hence by dynamics of lipoprotein secretion and clearance by the liver [30]. It was notable to find a positive correlation between decrease in plasma VLDL1-TG and improvement in CVD risk in the current study. As QRISK also improves in those who did not recover beta cell function it is unlikely that insulin secretion itself determined improvement in CVD risks. We have previously shown that muscle insulin resistance does not change during the first 6 months of weight loss although hepatic insulin sensitivity completely normalizes [4,22]. The latter is likely to be linked to the return to normal of hepatic lipid metabolism although we did not specifically assess this.

Plasma level of ApoB has been recently considered a more useful factor in determining CVD risk than the traditionally used LDL-cholesterol levels [31]. The large difference between responders and non-responders in the index of LDL particle size at 6 months is in line with effect of ApoB on CVD risk. ApoE function is important for the clearance of atherogenic lipoprotein remnants via binding to LDL receptors on the liver, thereby decreasing the risk of atherosclerosis and other CVD complications [9]. The lack of such clearance of lipoproteins remnants was found to be related to interference with the binding of ApoB/ApoE receptors via high expression of ApoC-III [32,33]. However, it was reported recently that ApoC-III promotes lipoprotein lipase (LPL) activity independent of ApoE-mediated clearance of lipoproteins [34]. There are conflicting reports whether plasma level of ApoE is related to CVD [35]. In the present study, plasma ApoE was lower in the long duration group and decreased after weight loss irrespective of remission. However, total plasma ApoE pool represents ApoE derived from VLDL, IDL, and chylomicrons. LDL, the most atherogenic of other lipoproteins, will eventually lose ApoE, and therefore plasma level of ApoE alone is probably insufficient indicator of CVD risk.

The changes in physiologic condition induced in this group of people with T2DM permits the potential utility of plasma biomarkers to be examined and for the relationships of biomarkers to change in body weight to be evaluated. Baseline GDF-15 but not FGF-21, leptin, nor adiponectin correlates with 10-year cardiovascular risk. FGF-21, GDF-15 and leptin all decrease and adiponectin increases with weight loss irrespective of remission. There are conflicting reports about the effect of dietary and surgical weight loss on FGF-21 level [36,37]. In mice, hepatic expression of FGF-21 was reported to enhance the cellular process that lead to the browning of white adipose tissues and selective expansion of subcutaneous adipose tissue [38,39].

The role of GDF-15 and FGF-21 on both T2DM and CVD has raised important questions about utility of these biomarkers and their possible mechanistic actions [15,16]. The association of GDF-15 with CVD in T2DM at baseline was confirmed, with novel data on GFD-15 changes after weight loss. However, there was no relationship between change in GDF-15 or FGF-21 levels and improvement in QRISK, suggesting that neither biomarker plays a mechanistic role or responsible for the changes in physiologic state.

Raised plasma levels of leptin in obesity and T2DM reflects leptin resistance, and relatively decrease response to leptin in regulating energy balance and cell metabolism [13,40]. However, any leptin effect on cardiovascular health is not completely understood [18,19,20]. In T2DM, leptin has been reported by some but not all studies to be a marker for CVD [18,19] and plasma leptin to adiponectin ratio has been described as a marker of atherogenicity [12,20]. It is known that leptin level decreases and adiponectin level increases following dietary induced weight loss in overweight non-diabetic individuals [41,42], and our data confirm that this is so also in T2DM. Adiponectin level increased also after surgical weight loss although the ratio of adiponectin/leptin was not a predictor of remission of T2DM after bariatric surgery [43] and the present data confirm this after dietary weight loss. However, the change in both leptin and adiponectin after dietary weight loss correlated with change in plasma HDL cholesterol.

The limitations of this study must be considered. Although small compared to most cardiovascular risk studies, the large effect size permits highly significant results. Secondly, only people of white European ethnicity were studied, reflecting the population of the North East of England, and specific studies are required to examine the biomarker relationships in other ethnicities [44]. Thirdly, our follow up period was short, and the study did not include a non-diabetic control group for comparison with the normal range in blood parameters and CVD risks. Fourthly, the study did not measure ApoC-III or lipoprotein (a), which are found to be risk factors for CVD events [33,45]. Finally, the algorithm used for measuring QRISK has limitation in terms of diabetes status in the absence of long-term outcome data on CVD during remission. Thus, those achieved remission of diabetes were considered free from diabetes, as there is no other option to describe the exact metabolic status of this group.

## 5. Conclusions

This study quantitates the major CVD beneficial effects of substantial dietary weight loss with remission of T2DM. In those who achieved remission, weight loss decreased substantially the 10-year cardiovascular risk, and decreased heart age by 15.0 years compared with 5.0 years in non-responders. Plasma levels of GDF-15 correlated with baseline risk of cardiovascular disease but did not provide an index of risk. The lack of predictive value of GDF-15, FGF-21, leptin and adiponectin will inform future research for better clinical management.

## Figures and Tables

**Table 1:** Clinical and metabolic characteristics of the studied groups at baseline and after intervention.

	Responders (*N* = 12)			Non-Responders (*N* = 17)		
	Baseline	2 Months	6 Months	Baseline	2 Months	6 Months
**Sex** (F/M)	4/8	-	-	10/7	-	-
**Age** (year)	52.0 ± 2.9	-	-	59.9 ± 2.1 #	-	-
**Diabetes Duration** (Year)	3.8 ± 1.0	-	-	9.8 ± 1.6 ##	-	-
**Weight** (kg)	99.8 ± 3.2	83.7 ± 3.0 ***	84.4 ± 3.2 ***	96.7 ± 3.9	83.8 ± 3.4 ***	84.8 ± 3.7 ***
**Fasting Insulin** (mU/L)	20.4 [12.4–25.0]	7.9 [7.1–11.3] **	7.6 [6.5–9.5] **	9.3 [5.7–11.7] ##	5.5 [3.4–8.4]	5.9 [4.2–9.5]
**Fasting Glucose** (mmol/L)	8.9 ± 0.7	5.1 ± 0.2 ***	6.2 ± 0.3 **	13.2 ± 0.6 ###	8.5 ± 0.8 ***##	9.4 ± 0.7 ***###
**HbA1c** (mmol/mol)	54.5 ± 3.7	39.8 ± 2.2 ***	41.4 ± 1.6 **	68.1 ± 3.2 #	62.8 ± 4.4 ###	61.6 ± 3.3 ###
**Total Fat** (%)	36.2 ± 1.9	30.1 ± 2.0 ***	31.5 ± 1.9 *	42.6 ± 2.2 #	37.2 ± 2.0 ***#	40.8 ± 2.5 ##
**Total TG** (mmol/L)	1.7 [1.4–2.2]	0.9 [0.7–1.1] **	1.2 [1.0–1.4] *	1.3 [1.2–1.8]	1.0 [0.7–1.1] **	1.2 [0.7–1.6]
**Liver Fat** (%)	10.6 [7.0–16.0]	2.1 [1.7–2.5] ***	1.7 [1.6–2.2] ***	7.3 [5.4–9.8]	2.2 [1.8–2.6] ***	2.0 [1.7–2.6] ***
**VLDL1-TG PR** (mg/kg/day)	411.0 ± 49.5	250.2 ± 37.1 **	257.8 ± 38.9 **	437.2 ± 32.4	314.7 ± 33.9 **	308.1 ± 30.4 **
**Fasting VLDL1-TG** (mmol/L)	0.60 [0.44–0.89]	0.32 [0.19–0.45] **	0.36 [0.25–0.45] **	0.43 [0.33–0.63]	0.26 [0.20–0.44] **	0.28 [ 0.17–0.42] **
**VLDL1-TG pool** (mg)	2322.5 [1496.4–3248.0]	991.7 [531.4–1438.8] ***	1038.4 [755.8–1343.2] **	1404.3 [929.3–2509.3]	770.5 [461.9–1391.8] **	607.8 [513.5–1493.5] **
**ApoB** (mg/mL)	1.6 [0.9–3.6]	1.7 [1.0–4.9]	2.3 [1.2–4.8]	1.1 [0.9–1.5]	1.1 [0.8–1.3]	1.0 [0.8–1.2] #
**ApoE** (µg/mL)	14.2 [9.3–17.0]	5.9 [5.0–10.1] **	5.1 [4.2–12.9] **	7.0 [5.6–10.4]	5.6 [3.5–8.2] *	5.1 [3.3–8.6] *
**Pancreas Fat** (%)	4.5 ± 0.3	4.0 ± 0.3*	3.7 ± 0.3 **	5.5 ± 0.8	5.5 ± 0.7	4.9 ± 0.6
**Total Cholesterol** (mmol/L)	4.7 ± 0.3	3.6 ± 0.2 **	4.2 ± 0.3 *	4.7 ± 0.3	3.7 ± 0.2 ***	4.2 ± 0.2 **
**HDL Cholesterol** (mmol/L)	1.1 ± 0.1	1.1 ± 0.1	1.4 ± 0.1*	1.3 ± 0.1 #	1.2 ± 0.1 *	1.5 ± 0.1
**Ratio HDL**/**Total Chol**.	4.5 ± 0.3	3.4 ± 0.3 *	3.3 ± 0.3 *	3.7 ± 0.3	3.2 ± 0.3	3.1 ± 0.3
**Leptin** (ng/mL)	34.4 [22.2–41.0]	13.3 [8.2–19.9] **	19.8 [8.5–25.7] **	46.1 [22.2–56.1]	14.7 [10.7–29.7] ***	18.3 [10.3–42.6] **
**Adiponectin** (µg/mL)	3.7 [3.0–4.4]	5.2 [3.6–5.7] *	5.3 [3.9–6.5] *	4.5 [3.7–5.8]	5.0 [4.3–6.7]	5.4 [4.2–7.2] **
**Leptin/Adiponectin** (ng/µg)	7.9 [6.4–14.8]	3.2 [1.3–6.3] **	3.9 [1.1–7.6] **	8.3 [5.2 –13.0]	4.1 [1.2–6.2] ***	3.9 [1.6 –9.7] ***
**10-Year QRISK** (%)	15.3 ± 3.4	5.2 ± 1.3 **	5.8 ± 1.6 ***	21.4 ± 2.9	14.6 ± 2.3 ***##	15.0 ± 2.0 ***###
**Heart Age** (year)	67.8 ± 2.9	51.8 ± 2.9 ***	52.7 ± 3.3 ***	74.6 ± 1.9	69.0 ± 2.1 ***###	69.9 ± 2.0 ***###

* *p* < 0.05, ** *p* < 0.01, *** *p* < 0.001 (vs. baseline). # *p* < 0.05, ## *p* < 0.01, ### *p* < 0.001 (vs. responders).

## Data Availability

The data are available on request from the corresponding author.

## Supplementary Materials

The following are available online at https://www.mdpi.com/article/10.3390/nu13051465/s1. Table S1: Clinical characteristics of short vs. long duration groups at baseline and after weight loss, Figure S1. Relationship between visceral fat, CVD risk, and plasma biomarkers at baseline, Figure S2. Correlation between change in CVD risk and change in plasma biomarkers.
