# Supplementary material for: Effect of Weight Loss by Low-Calorie Diet on Cardiovascular Health in Type 2 Diabetes: An Interventional Cohort Study
Source: Nutrients. 2021 Apr 26;13(5):1465. doi: 10.3390/nu13051465 (PMC8146720; doi:10.3390/nu13051465)
Supplement: Supplementary file 1 [file nutrients-13-01465-s001.zip › Supplementary Section.pdf]

nutrients MDPI

Supplementary Materials

Table S1. Clinical characteristics of short vs. long duration groups at baseline and after weight loss

	Short duration(<4 years) (*N*=15)			Long duration (>8 years) (*N*=14)		
	**Baseline**	**2 months**	**6 months**	**baseline**	**2 months**	**6 months**
**Sex** (F/M)	8/7	-	-	6/8	-	-
**Age** (year)	52.1±2.6	-	-	61.6±2.0##	-	-
**Weight** (kg)	99.0±3.7	84.5±3.5***	84.7±3.7***	96.9±3.8	83.0±3.2***	84.6±3.5***
**BMI** (kg/m^2)	34.1±0.8	29.1±0.9***	29.2±0.8***	34.4±1.2	29.4±1.1***	30.0±1.2***
**F. Insulin** (mU/L)	17.4[11.5–22.4]	7.9[7.1–11.5]**	6.2[10.6–4.4]**	7.0[5.7–11.6]##	5.4[3.6–7.9]#	5.9[4.2–7.6]
**F. Glucose** (mmol/L)	9.6±0.7	5.8±0.2***	7.0±0.3***	13.4±0.8##	8.4±1.1***#	9.3±1.0***#
**HbA1c** (mmol/moL)	55.1±2.4	43.6±2.0***	46.1±2.0***	70.3±4.1##	63.7±5.6##	61.2±4.5##
**Total Fat** (%)	39.3±2.0	33.6±2.0***	34.1±1.7***	40.6±2.6	35.1±2.5***	40.0±3.3
**Total TG** (mmol/L)	1.5[1.3–2.3]	0.9[0.7–1.1]**	1.3[1.1–1.6]**	1.3[1.2–1.7]	1.0[0.7–1.1]**	0.9[0.7–1.3]*
**Liver Fat** (%)	9.8[6.9–14.8]	2.1[1.8–2.9]***	1.7[1.6–2.5]***	7.3[4.8–8.4]	2.2[1.7–2.4]***	2.0[1.7–2.2]***
**Pancreas Fat** (%)	4.3±0.3	4.1±0.2	4.0±0.3	5.9±0.9	5.6±0.8	4.9±0.7*
**Total Chol.**(mmol/L)	4.6±0.2	3.6±0.2**	4.2±0.2*	4.8±0.3	3.7±0.3***	4.2±0.3**
**HDL Chol.** (mmol/L)	1.1±0.1	1.1±0.1	1.3±0.1*	1.4±0.1#	1.3±0.1#	1.5±0.1*
**Ratio Chol. Total/HDL**	4.4±0.3	3.5±0.3*	3.4±0.3***	3.7±0.4	3.1±0.4**	2.9±0.3***
**Leptin** (ng/ml)	39.1[29.2–58.8]	15.8[12.5–27.5]***	20.5[11.5–31.6]**	38.0[15.9–47.4]	12.2[8.3–17.6]***	16.0[9.1–36.3]**
**Adiponectin** (μg/mL)	3.7[3.5–4.4]	5.0[3.6–5.6]*	5.3[3.8–6.1]**	4.9[3.7–5.7]	5.5[4.5–7.0]*	5.9[4.2–7.4]*
**leptin/Adiponectin** (ng/μg)	8.8[6.4–21.1]	4.1[3.0–8.9]**	4.7[2.6–8.6]**	7.5[4.7–10.0]	1.6[1.1–5.2]**	2.4[1.4–9.1]**
**GDF-15** (ng/ml)	0.82±0.09	0.80±0.07	0.69±0.06*	1.19±0.14#	1.06±0.13	0.92±0.13*
**FGF-21** (ng/ml)	0.64[0.47–0.97]	0.57[0.40–0.71]*	0.41[0.25–0.56]**	0.79[0.51–1.32]	0.57[0.30–0.89]**	0.49[0.22–0.71]**
**VLDL1-TG PR** (mg/kg/day)	428.6±39.2	267.8±39.3**	295.3±37.5***	425.0±38.9	306.3±32.7**	278.6±30.7*
**Fasting VLDL1-TG** (mmol/l)	0.60[0.36–0.89]	0.39[0.21–0.50]**	0.40[0.23–0.45]**	0.43[0.38–0.57]	0.23[0.19–0.29]**	0.26[0.17–0.34]
**VLDL1-TG pool** (mg)	2370.9 [1082.5–3248.0]	1208.8 [488.6–1806.4]**	1236.5 [563.0–1432.8]**	1404.3 [1223.6–1951.5]	686.5 [479.8–864.9]**	696.9 [521.0–1061.8]*
**ApoB** (mg/ml)	1.1[6.6–20.3]	1.1[3.6–11.5]	1.2[3.3–11.1]	1.2[2.3–7.3]	1.2[1.0–7.0]	1.2[1.4–6.6]
**ApoE** (μg/ml)	15.6[9.6–17.6]	6.5[5.0–10.1]**	6.8[4.2–10.8]**	6.3[4.8–9.3] ##	4.8[3.4–6.4]*	4.4[3.2–7.0]
**10-Year QRISK** (%)	15.3±3.4	6.5±1.6**	6.6±1.5**	22.8±2.6#	15.2±2.6***##	16.1±2.3***##
**Heart Age** (year)	68.2±2.6	55.7±3.0***	56.3±3.1***	75.6±2.0#	68.6±2.7***##	69.7±2.6***##

*p<0.05, **p<0.01, ***p<0.001 (vs. baseline). #p<0.05, ##p<0.01, ###p<0.001 (vs. short duration).

nutrients

MDPI

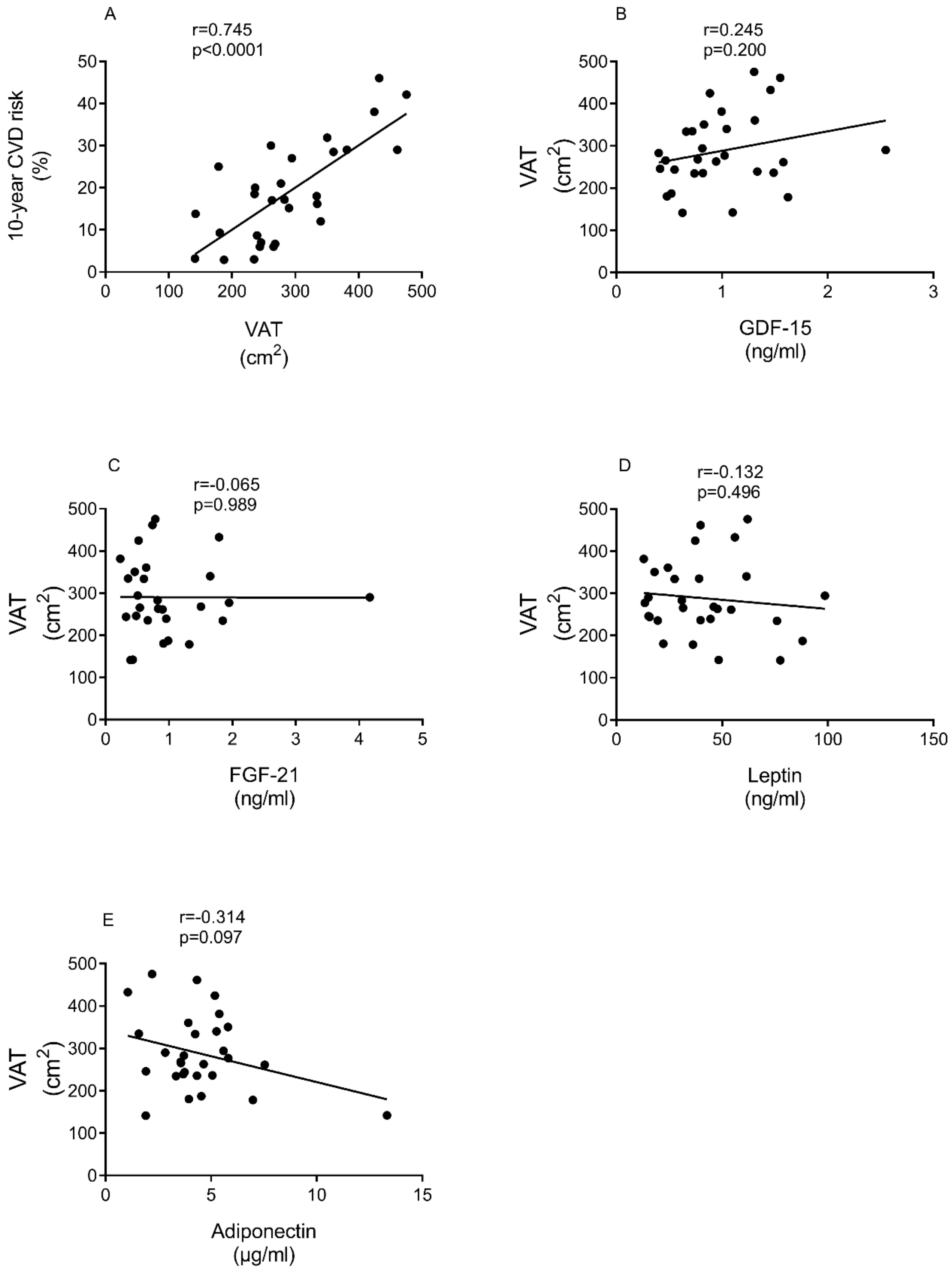

Figure S1. Relationship between visceral fat (VAT), CVD risk, and plasma biomarkers at baseline. A: 10-year CVD risk and VAT, B: VAT and GDF-15, C: VAT and FGF-21, D: VAT and Leptin, E: VAT and Adiponectin. There were 29 participants (12 responders/17 non-responders). Spearman Rho/Pearson correlations were used as appropriate.

nutrients MDPI

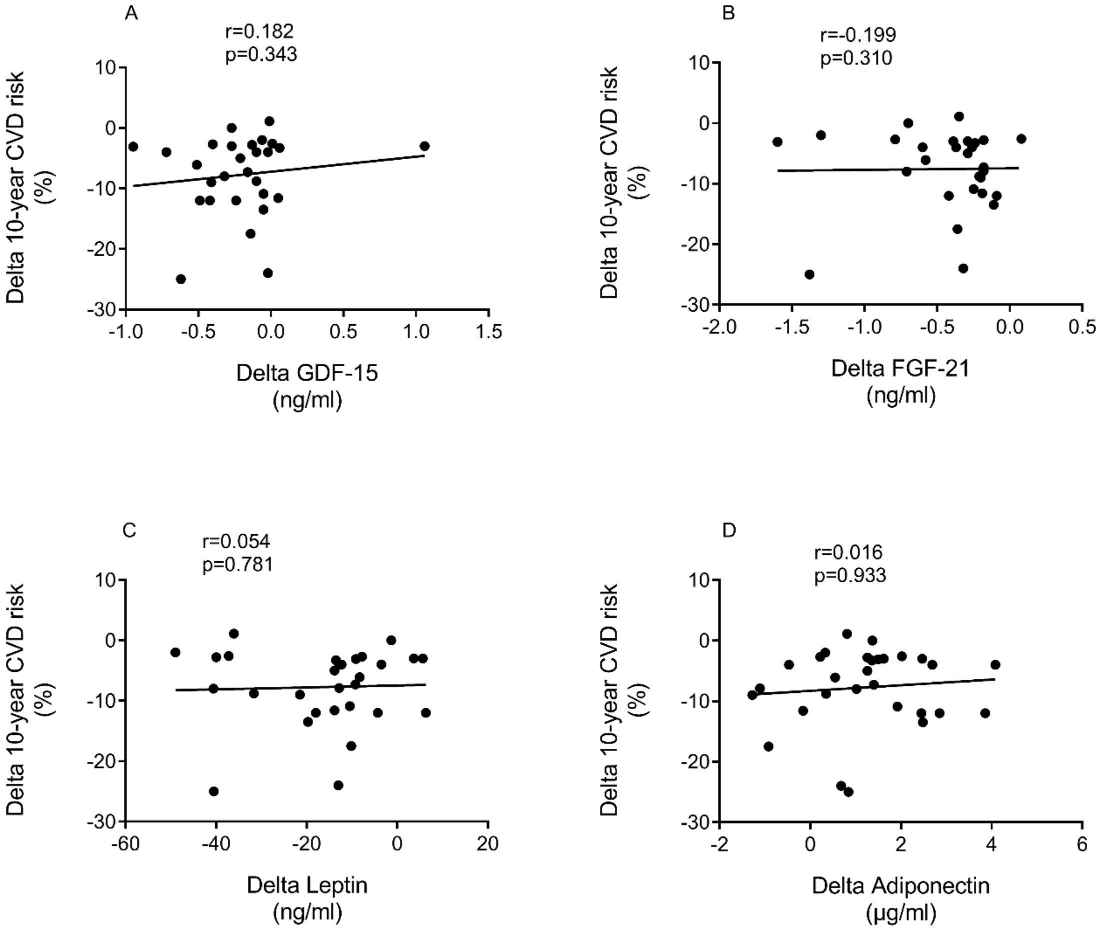

Figure S2. Correlation between change in CVD risk and change in plasma biomarkers. A: GDF-15, B: FGF-21, C: Leptin, D: Adiponectin. There were 29 participants (12 responders/17 non-responders). Spearman Rho/Pearson correlations were used as appropriate. Correlations were done for the change from baseline at 6 months.
